# Fiber-reinforced composite restorations in endodontically treated teeth under functional loading

**DOI:** 10.6026/973206300222706

**Published:** 2026-05-31

**Authors:** Pranav D Patil, T Rajsundar, E Amuthavalli, U Punitha Gnana Selvi, Pradnya Jadhav, Manini Soni

**Affiliations:** 1 Department of Conservative Dentistry and Endodontics, Bharati Vidyapeeth (Deemed to be University) Dental college and Hospital, Sangli, Maharashtra, India; 2 Department of Periodontology, Tamil Nadu government dental college and hospital, Satellite peripheral unit, Tambaram, Chennai, Tamil Nadu, India; 3 Department of Dental Surgery, KAPV Government Medical College, Trichy, Tamil Nadu, Indiasd; 4 Department of Public Health Dentistry, Government Dental College and Hospital, Chhatrapati Sambhajinagar, India; 5 Department of Conservative Dentistry and Endodontics, Goenka Research Institute of Dental Sciences, Gandhinagar District, Near G.G.S, Piplaj, Gujarat, India

**Keywords:** Fiber-reinforced composite (FRC), endodontically treated teeth, fracture resistance, functional loading, marginal adaptation, microleakage

## Abstract

Endodontically treated teeth remain highly susceptible to fracture due to significant loss of structural integrity and optimal restorative material selection remains a clinical challenge. Therefore, it is of interest to evaluate fracture resistance, marginal adaptation and microleakage of fiber-reinforced composite (FRC) restorations compared with conventional composite and metal-ceramic crowns under simulated functional loading. Ninety extracted human premolars were randomly assigned to three groups and subjected to cyclic loading (200 N, 500,000 cycles) using a chewing simulator. FRC restorations demonstrated significantly higher fracture resistance (987.4 ± 62.3 N) compared to conventional composite (743.2 ± 58.1 N) and showed comparable marginal adaptation to metal- ceramic crowns (p<0.001). Fiber-reinforced composites may provide a biomechanically superior and clinically viable alternative for restoring endodontically treated teeth, particularly in high load-bearing posterior regions.

## Background:

One of the most difficult situations in the field of restorative dentistry is the treatment of endodontically treated teeth, the main reason of which is the loss of pulpal tissue and mechanical preparation of the root canals, which leads to a significant loss of coronal and radicular dentin, making the tooth structure a significant compromise [1]. The decreased moisture values of dentin after devitalization have further changed the mechanical values of the tooth, decreasing its toughness and thereby becoming more vulnerable to disastrous fracture under functional loads [2]. These biological and mechanical alterations require a close attention to the restorative methodology so that the long-term clinical success and maintenance of the rest of the tooth structure could be achieved. In the past, the cast metal posts and cores were replaced by full-coverage metal- ceramic crowns, which were regarded as the gold standard of restoration of teeth that had been endodontically treated [3]. Nevertheless, the prevailing clinical interest in tooth-colored, esthetic restorations and increased awareness of the stress-concentrating properties of rigid metallic restorations has met the shift in clinical focus towards more biomechanically favorable alternatives [4]. The posts made with fiber-reinforced composite have an elastic modulus of approximately 18-20 Gpa and have shown the ability to distribute functional loads in a more even way along the root and in so doing minimizing the occurrence of catastrophic root fractures [5]. Coronal restorative materials, which are fiber-reinforced composites, are a novel field of research, as opposed to post systems. These resin based materials also have the added benefit of being able to use glass, polyethylene, or quartz fibers in a resin matrix, which gives them better flexural strength, fracture toughness and better distribution of stress than traditional particulate filled composites [6]. It has been demonstrated that the mechanism of fiber reinforcement suppresses crack propagation through deflection and energy dissipation at the fiber-matrix interface, which is especially ideal in high stress conditions like the posterior dentition [7]. Recent research has given promising figures concerning the utilization of fiber-reinforced systems in different restorative uses. Research has demonstrated that short fiber reinforcement into bulk-fill composites is a great way of enhancing the fracture toughness and fatigue load resistance than the traditional composite types [8]. Also, the marginal integrity of FRC restorations has been tested in thermocycling and load cycling conditions and the results indicated that it has a better sealing capacity than that of monolithic composite restorations [9]. The idea of biomimetic restoration, which entails the imitation of the natural mechanical demeanor of natural tooth structure, has likewise become popular as a guiding principle in the choice of restoration materials in degraded teeth [10]. Nevertheless, there are still a number of gaps in the literature. Most of the research has concerned FRC post systems but not FRC coronal restorative, so little is known about the direct coronal restorative of fiber-reinforced composites in endodontically-treated teeth [11]. Moreover, the majority of laboratory studies have utilized the static loading conditions, which do not well recreate the complex and cyclical conditions of masticatory forces in clinical practice [12]. Therefore, it is of interest to evaluate and compare the mechanical performance and marginal integrity of fiber-reinforced composite restorations with conventional composite and metal-ceramic crowns under simulated functional loading.

## Materials and Methods:

## Study design and sample size:

This study was designed as a controlled, *in vitro* comparative laboratory investigation. A total of 90 freshly extracted human maxillary premolars were collected. Sample size calculation was performed using G*Power software (version 3.1.9.7), with an effect size of 0.40, alpha level of 0.05 and power of 0.80, yielding a minimum of 28 specimens per group. Ninety specimens were used to provide an adequate buffer for potential specimen loss and to increase statistical power.

## Inclusion and exclusion criteria:

eeth were included if they were fully erupted maxillary first or second premolars with complete root formation, single or double roots of similar dimensions and absence of visible cracks, caries, or previous restorations. Teeth were excluded if they showed signs of root resorption, calcified canals, developmental anomalies, or if they had been stored for more than six months prior to the study. All extracted teeth were stored in 0.1% thymol solution at 4°C until use and were used within three months of extraction.

## Specimen preparation and grouping:

All teeth underwent standardized endodontic treatment performed by a single calibrated operator. Access cavities were prepared using high-speed diamond burs under copious water irrigation. Root canals were instrumented using a rotary nickel-titanium system (ProTaper Gold, Dentsply Sirona) to an apical size of F3, with irrigation performed using 2.5% sodium hypochlorite and 17% EDTA. Obturation was completed using warm vertical compaction with gutta-percha and AH Plus sealer (Dentsply DeTrey). Following obturation, standardized MOD (mesio- occluso-distal) cavities were prepared to simulate typical clinical tooth structure loss, with a standardized bucco-lingual width of 4.0 mm and an isthmus depth of 3.0 mm.

Specimens were randomly allocated into three equal groups (n = 30 per group) using a computer-generated randomization table:

[1] Group 1 (FRC): Restoration using fiber-reinforced composite (EverX Posterior, GC Corporation) with a 2 mm base layer, covered by a conventional nanohybrid composite (G-aenial Posterior, GC Corporation).

[2] Group 2 (CC): Restoration using conventional nanohybrid composite alone (G-aenial Posterior, GC Corporation) in an incremental layering technique.

[3] Group 3 (MCC): Full-coverage metal-ceramic crowns fabricated by a certified dental laboratory using a cobalt-chromium framework veneered with feldspathic porcelain, luted with resin-modified glass ionomer cement (RelyX Luting Plus, 3M ESPE).

All adhesive procedures were performed using a universal adhesive system (Scotchbond Universal, 3M ESPE) in self-etch mode, with light curing performed using a LED curing unit (Elipar DeepCure-S, 3M ESPE) at an intensity of ≥1200 mW/cm^2^.

## Cyclic loading:

Following restoration, all specimens were subjected to thermocycling (5°C to 55°C, 500 cycles, 30-second dwell time) to simulate thermal stresses encountered intraorally. Specimens were then mounted in acrylic resin blocks with the cemento-enamel junction positioned 2 mm above the resin surface, simulating physiological bone support. Cyclic loading was performed using a chewing simulator (CS-4.8, SD Mechatronik, Germany) at a load of 200 N applied vertically to the central fossa at a frequency of 1.6 Hz for 500,000 cycles, simulating approximately two years of masticatory function.

## Assessment parameters:

[1] Fracture resistance: Following cyclic loading, specimens were subjected to compressive loading in a universal testing machine (Zwick/Roell Z010) at a crosshead speed of 1 mm/min until fracture occurred. Maximum load at fracture was recorded in Newtons (N). Fracture mode was classified as repairable (coronal fracture) or catastrophic (subgingival or root fracture).

[2] Marginal adaptation: Marginal adaptation was evaluated before and after cyclic loading using a scanning electron microscope (SEM, JEOL JSM-6510, Japan) at x 500 magnifications. Marginal gaps were measured at four standardized points per specimen and rated using a modified USPHS (United States Public Health Service) criterion: Score 0 (no gap), Score 1 (gap < 50 µm), Score 2 (gap 50-150 µm), Score 3 (gap > 150 µm).

[3] Microleakage: Specimens were sealed with nail varnish to within 1 mm of the cavity margins and immersed in 2% methylene blue dye solution for 24 hours. Following longitudinal sectioning, dye penetration was scored on a scale of 0-3 under a stereomicroscope (Zeiss Stemi 508) at x20 magnification.

## Statistical analysis:

Data were analyzed using IBM SPSS Statistics version 27.0. Normality of continuous data was assessed using the Shapiro-Wilk test. Fracture resistance data (normally distributed) were analyzed using one-way ANOVA followed by Tukey's post-hoc test. Marginal adaptation and microleakage scores (ordinal data) were analyzed using the Kruskal-Wallis test followed by Dunn's post-hoc test with Bonferroni correction. Fracture mode distribution was analyzed using the Chi-square test. Statistical significance was set at p < 0.05.

## Results:

The mean fracture resistance values following cyclic loading are presented in Table 1. The FRC group demonstrated the highest mean fracture resistance (987.4 ± 62.3 N); followed by the MCC group (1,024.7 ± 78.6 N) and the CC group (743.2 ± 58.1 N). One-way ANOVA revealed a statistically significant overall difference among groups (F = 89.34, p < 0.001). Tukey's post-hoc analysis confirmed that the CC group was significantly different from both the FRC (p < 0.001) and MCC groups (p < 0.001), while no statistically significant difference was observed between the FRC and MCC groups (p = 0.082). Regarding fracture mode, the FRC group exhibited the highest proportion of repairable fractures (80%), compared to 56.7% in the MCC group and 63.3% in the CC group. The difference in fracture mode distribution was statistically significant (Chi-square = 8.74, p = 0.013). Marginal adaptation scores before and after cyclic loading are presented in Table 2. Prior to loading, all three groups demonstrated predominantly Score 0 and Score 1 marginal adaptation. Following cyclic loading, the FRC group maintained superior marginal integrity compared to the CC group, with a statistically significant difference (p = 0.003). The MCC group demonstrated the most favorable post-loading marginal adaptation scores. Kruskal-Wallis analysis revealed a significant overall group difference in post-loading marginal adaptation (H = 18.43, p < 0.001). Post-hoc analysis confirmed significant differences between FRC and CC groups (p = 0.003) and between MCC and CC groups (p < 0.001), but not between FRC and MCC groups (p = 0.214). Microleakage scores are presented in Table 3. The FRC group demonstrated significantly lower microleakage scores compared to the CC group (p < 0.001), with 60% of FRC specimens scoring 0 and only 3.3% scoring 3. The CC group exhibited the highest microleakage values, with 20% of specimens scoring 3. The MCC group showed the lowest overall microleakage, with 73.3% scoring 0. Kruskal-Wallis analysis confirmed a statistically significant group difference (H = 22.17, p < 0.001). Post-hoc analysis revealed significant differences between FRC and CC (p < 0.001), MCC and CC (p < 0.001), but no significant difference between FRC and MCC (p = 0.178).

## Discussion:

The results of the current study give valuable ideas about the clinical applicability of fiber-reinforced composites as endodontically treated tooth restorative materials under functional loading. The key observation, i.e., that the values of the fracture resistance to FRC restorations were statistically equal to those of metal-ceramic crowns and far exceeded the results of traditional composites, confirms the theoretical background of the fiber reinforcement of dental materials and are confirmed by an accumulating body of experimental results. This high FRC group fracture resistance as compared to that of the CC group is explained by the special mechanism of stress- distribution that the embedded glass fibers impart. The fiber-matrix interface in the EverX Posterior material enables deflection and energy absorption of cracking that occurs by processes of fiber pullout and debonding which are well-developed toughening mechanisms in composite materials [6]. This ability to prevent crack propagation is especially noticeable in endodontically treated teeth, in which the underlying dentin substrate is more brittle and possesses lower elastic modulus than the vital teeth [7]. A notable conclusion of the present research is the considerably larger percentage of repairable types of fractures that were found in the FRC group (80 percent) than in the MCC group (56.7 percent). This finding has significant clinical implications, in that, because repairable fractures, which typically involve the coronal part of the tooth, will allow later restorative placement, catastrophic fractures of the root usually require tooth removal. This productive failure mode of FRC restorations is correlated with the reports that biomimetic restorations with fiber-reinforced layers are closer in deformation features to the natural enamel-dentin complexes that do not suffer any damage [10]. The high fracture resistance of rigid metallic structure of metal-ceramic crowns is unfavorable, as it concentrates the stresses on the crown-root interface and these results in the potential occurrence of unfavorable root fractures in extreme loading conditions [13]. The marginal adaptation findings of the present study are congruent with the already published data indicating that FRC restorations have an acceptable marginal integrity in cyclic loading conditions [9]. The decreasing margins of the Score 0 after loading in the FRC group (76.7 to 56.7) also shows that to some extent, there is some marginal degradation that occurs in any resin based restorative system because of polymerization shrinkage, viscoelastic degradation under loading and hydrolytic degradation at the resin-dentin interface [1]. Nevertheless, the much higher preservation of marginal integrity in the FRC group than the CC supports the existence of fiber reinforcement to restrain restoration deformation and resulting marginal gap development. Its microleakage results also support the clinical applicability of the FRC approach. One main etiological cause of secondary caries, marginal discoloration and post-operative sensitivity which undermine the survival of restorations in endodontically treated teeth is microleakage at the restoration tooth interface [15]. The mean dye penetration scores of FRC group is much lower than that of the CC group indicating that the mechanical support of the fiber-reinforced base layer can decrease interfacial stress at the adhesive interface and this keeps the resin-dentin bond intact in the cycles of loading. This observation is proven by the studies which have shown that restorations with a short fiber composite base cause less stress transmission to the wall of cavity in case of polymerization compared to conventional composites only [8]. It is not surprising that the MCC group performs best in the current study, with the greatest absolute fracture resistance and the lowest microleakage, as the mechanical durability of metal-ceramic crowns has been well already established in clinical practice. Nevertheless, the similar performance of FRC restorations in a variety of parameters and a clear benefit in terms of repairable fracture modes make FRC an option of a conservative alternative, especially in clinical cases where preservation of maximum tooth structure is a preferable factor [11]. Direct restoration conservation saves the massive reduction of axia and occlusiveness necessary to prepare a crown, which in turn has been linked to pulpal damage and further structural debilitation [4]. The shortcomings of this research must be taken into account. Being an *in vitro* study, the simulation of intraoral conditions, such as the complicated biochemical environment of saliva, the multidirectional character of masticatory forces and patient-specific factors, is an inherently flawed issue. The biomechanical complexity of clinical masticatory loading is not entirely recapitulated by the use of a single-cusp loading at a standardized angulation [12]. Also, the relatively short simulation time of 500,000 cycles, which is a scaled version of two years in service, may not be sufficient to focus on long term degradation effects of the total clinical lifespan of the restorations. Also, the experiment was conducted only on maxilla premolars and the findings might not be directly transferred to other types of teeth, especially the molars, which experience significantly greater occlusal forces [5]. Long-term prospective randomized clinical trials should be done in future studies to prove such laboratory findings in real clinical conditions. Research that also involves more fiber-reinforced composite systems, such as short fiber-reinforced bulk-fill composites and polyethylene fiber-reinforced systems, would add additional evidence to the body of research. Combination of digital optical coherence tomography to monitor marginal adaptability real-time and the finite element analysis in stresses distributions would also offer a complementary mechanistic insight into FRC performance in weakened teeth [16].

## Conclusion:

Fiber-reinforced composite restorations showed superior fracture resistance, marginal adaptation and reduced microleakage compared to conventional composites in endodontically treated premolars under functional loading. Although metal-ceramic crowns showed higher absolute fracture resistance, they were associated with more catastrophic fracture patterns. Fiber-reinforced composites may serve as a biomechanically favorable and tooth-conserving alternative, warranting further validation through long-term clinical studies.

## Figures and Tables

**Table 1:** Fracture resistance values (N) and fracture mode distribution following cyclic loading

**Group**	**n**	**Mean ±SD (N)**	**Min-Max (N)**	**Repairable Fractures (%)**	**Catastrophic Fractures (%)**	p-value*
FRC	30	987.4 ±62.3	841-1,098	80	20	<0.001
CC	30	743.2 ±58.1	618-852	63.3	36.7	
MCC	30	1,024.7 ±78.6	876-1,187	56.7	43.3	
*One-way ANOVA;
Tukey's post-hoc: FRC vs. CC,
p < 0.001;
MCC vs. CC, p < 0.001;
FRC vs. MCC, p = 0.082.
FRC = Fiber-Reinforced Composite;
CC = Conventional Composite;
MCC = Metal-Ceramic Crown.

**Table 2:** Marginal adaptation score distribution before and after cyclic loading (%)

**Group**	**Assessment Point**	**Score 0 (%)**	**Score 1 (%)**	**Score 2 (%)**	**Score 3 (%)**	p-value*
FRC	Before Loading	76.7	20	3.3	0	0.003
FRC	After Loading	56.7	30	10	3.3	
CC	Before Loading	73.3	20	6.7	0	
CC	After Loading	33.3	33.3	23.3	10	
MCC	Before Loading	80	16.7	3.3	0	
MCC	After Loading	66.7	26.7	6.7	0	
*Kruskal-Wallis test;
post-hoc Dunn's with Bonferroni
correction for post-loading scores.
Score 0: no gap;
Score 1: gap < 50 µm;
Score 2: gap 50-150 µm;
Score 3: gap > 150 µm.

**Table 3:** Microleakage score distribution among study Groups (%)

**Group**	**Score 0 (%)**	**Score 1 (%)**	**Score 2 (%)**	**Score 3 (%)**	**Median Score**	p-value*
FRC	60	23.3	13.3	3.3	0	< 0.001
CC	23.3	30	26.7	20	2	
MCC	73.3	20	6.7	0	0	
Kruskal-Wallis test;
post-hoc Dunn's with
Bonferroni correction.
Score 0: no leakage;
Score 1: penetration into enamel margin;
Score 2: penetration into dentin margin;
Score 3: penetration to axial wall.
